# Relation of the lumbosacral trunk to the sacro-iliac joint

**DOI:** 10.1038/s41598-021-99851-3

**Published:** 2021-10-12

**Authors:** Peter Grechenig, Christoph Grechenig, Gloria Hohenberger, Marco Johannes Maier, Georg Lipnik, Angelika Schwarz, Theresa di Vora, Axel Gänsslen

**Affiliations:** 1 Department of Orthopaedics and Traumatology, Paracelsus Medical University, Müllner Hauptstraße 48, 5020 Salzburg, Austria; 2 Division of Macroscopic and Clinical Anatomy, Medical University of Graz, Harrachgasse 21, 8010 Graz, Austria; 3 Department of Ophthalmology, Medical University of Vienna, Währinger Gürtel 18-20, 1090 Vienna, Austria; 4 Department of Trauma Surgery, State Hospital Feldbach-Fürstenfeld, Ottokar-Kernstock-Straße 18, 8330 Feldbach, Austria; 5 Unaffiliated, Vienna, Austria; 6 AUVA Trauma Hospital Styria|Graz, Göstinger Str. 24, 8020 Graz, Austria; 7 Medical University of Graz, Auenbruggerplatz 2, 8036 Graz, Austria; 8 Clinic for Trauma Surgery, Orthopaedics and Hand Surgery, Klinikum Wolfsburg, Sauerbruchstr. 7, 38440 Wolfsburg, Germany

**Keywords:** Anatomy, Musculoskeletal system

## Abstract

This study aims to evaluate the relation between the lumbosacral trunk (LT) and the sacro-iliac joint (SIJ). In forty anatomic specimens (hemipelves) a classical antero-lateral approach to the SIJ was performed. The SIJ was marked at the linea terminalis (reference point A). Reference point B was situated at the upper edge of the interosseous sacro-iliac ligament. The length of the SIJ (distance A to B) and the distance between point A and the ventral branch of the fourth (L4) and fifth (L5) lumbar spinal nerves at the linea terminalis were measured. The SIJ had a mean length of 58.0 mm. The ventral branch of L5 was located closer to the SIJ in very long SIJs (mean length: ≥ 6.5 cm; mean distance: 9.8 mm) compared to very short joints (≤ 5 mm; mean distance: 11.3 mm). For the ventral branch of L4, very long SIJs had a mean distance of 7 mm and very short joints an average distance of 9.7 mm between point A and the nerve branch. A safe zone of approximately 1 cm to 2 cm (anterior to posterior) is present on the sacral surface (lateral to medial) for safe fixation of plates during anterior plate stabilization of the SIJ. Pelves with a shorter dorsoventral diameter of the most superior part of the SIJ apparently give more space for inserting plates.

## Introduction

The lumbosacral trunk (LT) represents a link between the lumbar and the sacral plexus^1^. It is formed by the caudal portion of the ventral ramus of the fourth and the complete ventral ramus of the fifth lumbar spine nerve. The LT descends medially to the psoas major muscle, reaches the superior aspect of the ala of sacrum, crosses the linea terminalis and joins the ventral branch of the first sacral spinal nerve at the level of the first anterior sacral foramen^2,3^.

The surgical relevance of the topographical relation between the LT and the sacro-iliac joint (SIJ) becomes obvious during open reduction and internal fixation (ORIF) of injuries of the SIJ. Especially, techniques of anterior plate stabilization in pure SIJ dislocations or stabilization of fracture dislocations of the SIJ can potentially harm the LT^4,5^. Typically, during exposure of the superolateral aspect of the sacrum, the LT is at risk^6^. Its distance to the anterior SIJ is reported to be only 10 mm^7^.

The LT is closely located to the superior anterior bony surface. Thus, vertical shear fractures in this area can cause stretching injuries^8^.

Only one singular analysis by Ebraheim et al.^7^, dealing with surgical anatomy of the LT relative to the SIJ during the anterior approach to the SIJ is available. Authors reported a mean distance of 23 mm and 26 mm to the ventral branches of L4 and L5 at the height of 4 cm proximal to the linea terminalis at the level of the anterior wall of the SIJ, while at the junction between the false and true pelvis a mean distance of 10 mm was observed^7^. Comparable values were reported in a recent combined CT and anatomical analysis by Bai et al.^9^.

A close relationship of the LT to the bone surface (0.1 mm) of the ala of sacrum at the level of the linea terminalis was reported by Mirkovic et al.^2^. Within the true pelvis, the internal iliac artery and the LT are as close as 5 mm to the anterior sacral cortex.

Using the antero-lateral approach in SIJ (fracture-)dislocation for plate stabilization, anatomical dissections revealed that the LT can nearly always be visualized^10^. Insertion of retractors to visualize the SIJ intraoperatively may additionally harm the LT.

Therefore, this anatomical study aimed to correlate the distance between the ventral rami of the fourth and fifth lumbar spinal nerves forming the LT with the length of the anterior wall of the SIJ.

## Material and methods

Dissection was performed on 40 anatomical specimens of hemipelves obtained from 38 whole pelves, embalmed using Thiel’s method^11^. This special embalming technique provides a life-like model through the preservation of the original tissue color, consistency and degree of transparency. None of the specimens showed signs of malformations or prior interventions in the area of interest. The anatomical specimens were donated to the Medical University of Graz. During their lifetime, donors had given their written informed consent to participate in anatomical studies.

### Dissection

Dissection was performed in the supine position. After removal of the bowel content from the abdominal and pelvic cavities, the iliac muscle was released from its origin by blunt dissection and the nerves of the lumbar plexus were identified, including the femoral nerve (FN), the obturator nerve (ON) and the LT.

**Figure 1:**
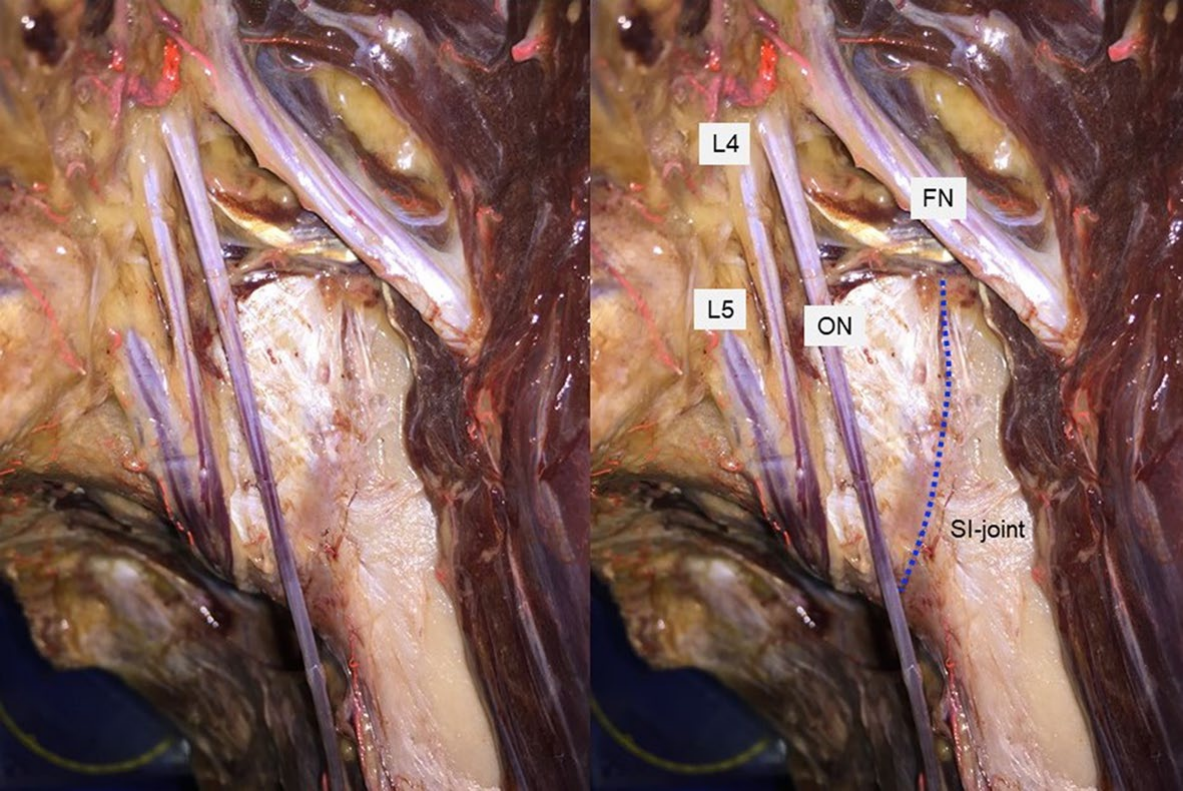
Dissected lumbosacral trunk in a left Thiel fixated hemipelvis with the additional part of the lumbar plexus (*FN* femoral nerve, *L4* ventral branch of fourth lumbar spinal nerve, *L5* ventral branch of fifth lumbar spinal nerve, *ON* obturator nerve).

For better visualization, parts of the iliopsoas muscle, the external and internal iliac vessels and parts of the FN and ON were removed (Fig. 1). A blunt dissection was performed to release the structures covering the sacroiliac joint. The sacro-iliac ligaments were partially removed for adequate visualization of the upper course of the SIJ from inside the true pelvis along its course lateral to the upper sacrum.

Further dissection was performed on the anterior surface of the sacrum to visualize the anterior rami of the sacral plexus.

### Measurements

The superior part of the fibrous capsule covering the SIJ was marked with a cannula at the intersection with the linea terminalis (reference point A), and at the posterior–superior edge of the interosseous sacro-iliac ligament (reference point B).

The length of the SIJ was measured as the straight distance between reference point A and B as the true length is highly variable due to a usually slightly curved course of the superior joint line. All distances were measured with a digital caliper and in millimeters.

First, the distance between reference point A and the lateral border of the ventral L4 and L5 rami at the linea terminalis (Inlet plane) was measured.

**Figure 2:**
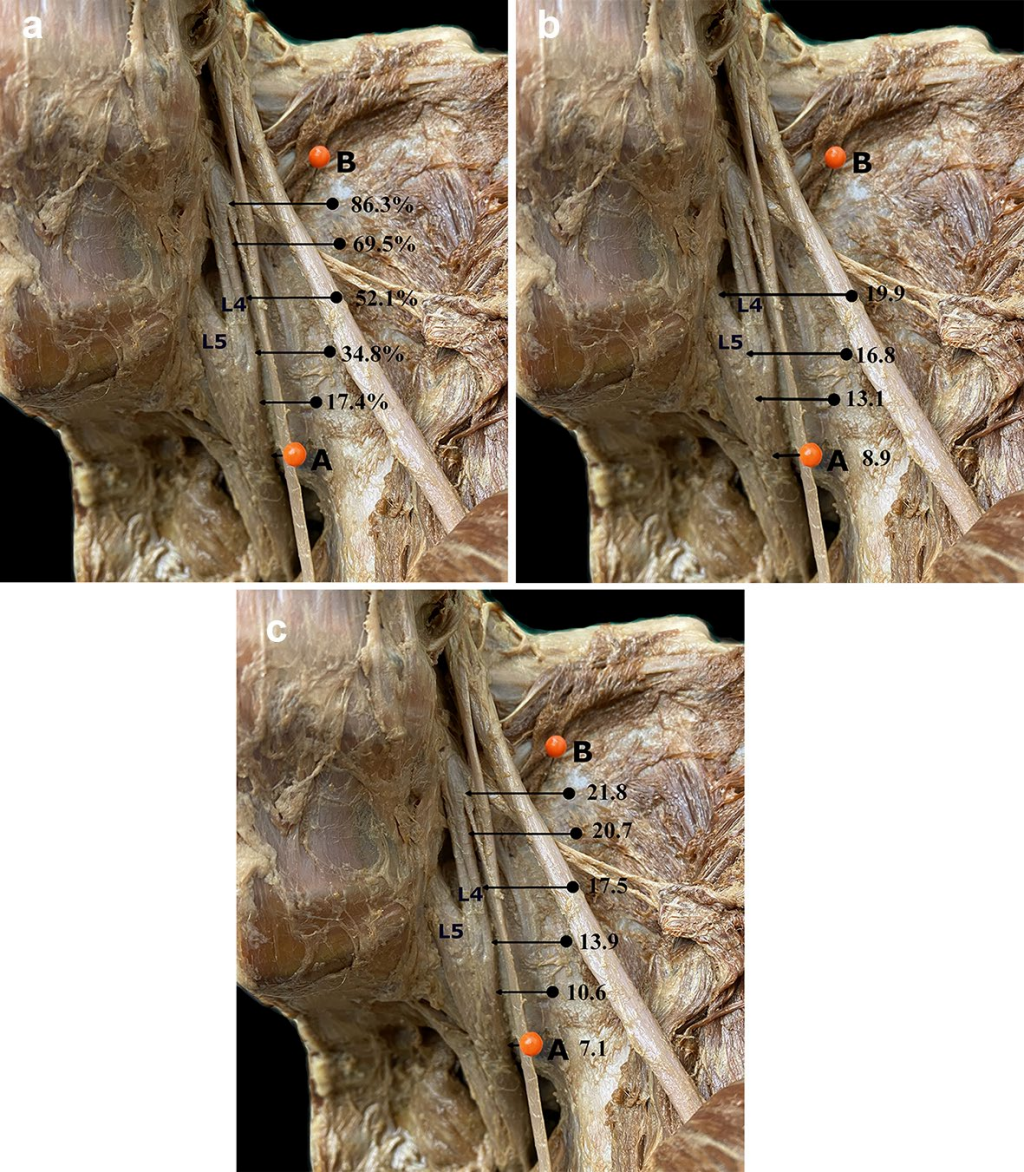
(**a**) Measurement pattern. Depicted are the respective measurement points as percentages with regard to the mean total length of the anterior wall of the sacro-iliac joint on a left hemipelvis embalmed using Thiel’s solution (*A* Marking of the superior part of the fibrous capsule covering the sacro-iliac joint at its intersection with the linea terminalis, *B* marking of the joint capsule at the posterior-superior edge of the interosseous sacro-iliac ligament, *L4* ventral branch of fourth lumbar spinal nerve, *L5* ventral branch of fifth lumbar spinal nerve). Note the marked difference in tissue color and preservation when compared to Fig. 1. (**b**) Depiction of the mean distances between the ventral ramus of the fifth lumbar spinal nerve (L5) and the anterior wall of the sacro-iliac joint at the respective measurement points in millimeters. (**c**) Means of the intervals between the anterior wall of the sacro-iliac joint and the ventral branch of fourth lumbar spinal nerve (L4) regarding the total collective. Data are presented in millimeters.

Starting from reference point A, along the true superior SIJ plane, the distances between the joint line and the lateral border of L4 and L5 were measured in 1 cm increments perpendicular to the joint line starting anterior in a posterior direction. These intervals were chosen due to their convenient applicability during surgery. For better standardization, these 1 cm subsections were evaluated as percentages of the individual length of the superior aspect of the SIJ (Fig. 2a).

Additionally, the distance from reference point A to the union of L4 and L5 was measured.

Starting from reference point A, 1 cm distal to the linea terminalis (Inlet plane) in a longitudinal direction the horizontal distance to the LT was measured.

### Data analysis

The collected data were analyzed in the statistical software R^12^. To assess differences between side and sex, Mann–Whitney U-test were employed. Concerning descriptive statistics, continuous variables are presented as mean and standard deviation (SD), minimum and maximum, categorical data as frequencies and percentages.

### Compliance with ethical standards

All investigated anatomical specimens were donated to the Division of Macroscopic and Clinical Anatomy of the Medical University Graz under the approval of the Anatomical Donation Program of the Medical University of Graz and according to the Austrian law for donations. Additionally, the study was performed in accordance with the relevant guidelines and regulations.

## Results

The hemipelves were gained from 38 body donors (15 female and 23 male donors) with a mean age of 81.6 years (SD: 8.4; range 56–96). Twenty left and 20 right hemipelves were dissected. Dissections were performed unilaterally in 36 and bilaterally in two specimens.

The SIJ had a mean length of 58.0 mm (SD: 0.5; range 4.5–6.9).

The average distance between point A and the union of L4 and L5 was 10.5 mm (SD: 2.6; range 5.5–17.5). In nearly all anatomic specimens the union occurred within the true pelvis at the anterior sacral surface.

The distances between the measurement points in 1 cm increments from reference point A, orientated posteriorly, and the lateral borders of L4 and L5 are presented in Table 1. In relation to the total length of the superior aspect of the SIJ, the measurement points were at a mean of 17.4% (1 cm distance), 34.8% (2 cm distance), 52.1% (3 cm distance), 69.5% (4 cm distance) and 86.3% (5 cm distance).

**Table 1:** Details on distances (in millimeters) between the SIJ and the lateral borders of the ventral branches of L4 and L5.

	Point A	17.4%	34.8%	52.1%	69.5%	86.3%
**L4**						
Mean	7.1	10.6	13.9	17.5	20.7	21.8
SD	2.3	2.3	3.0	3.9	5.1	2.9
Minimum	1.9	3.9	6.5	11.5	14.1	17.3
Maximum	11.9	16.7	24.4	31.6	38.9	26.5
Hemipelves	40	40	40	40	40	13

	Point A	17.4%	34.8%	52.1%		
**L5**						
Mean	8.9	13.1	16.8	19.9		
SD	2.6	2.5	3.5	5.1		
Minimum	3.2	5.2	8.9	13.2		
Maximum	15.8	19.1	28.7	38.3		
Hemipelves	40	40	40	29		

Point A represents the basic reference point at the linea terminalis.

Percentages represent the 1 cm measurement points relative to the total length of the SIJ starting from reference point A in a posterior direction (Inlet plane).

As the ventral ramus of L4 exits from its respective intervertebral foramen more superior when compared to L5, it was found to descend more distant to the SIJ (see Table 1).

### Measurements regarding the ventral ramus of L5

Measurements of the distance from reference point A at the linea terminalis to the ventral branch of L5 and the perpendicular distance at 17.4%, 34.8%, 52.1% were possible in 40, 40 and 29 cases, respectively. The corresponding distances were 8.9 mm, 13.1 mm, 16.8 mm and 19.9 mm (Fig. 2b).

Considering the measured length of the SIJ, 13 hemipelves presented with a length between 4.5 cm and 5.5 cm (mean: 5.2 cm) and were therefore ranked as pelves with a ‟shorter” SIJ. Thirteen hemipelves showed a length ≥ 6 cm (range 6.1–6.9 cm; mean: 6.4 cm) and were classified as pelves with a ‟longer” SIJ. Pelves with a ‟shorter” SIJ showed the respective mean distances of 9.1 mm (from reference point A), 13,7 mm (17.4%), 17,9 mm (34.8%) and 21 mm (52.1%). In comparison ‟longer” SIJs had mean intervals of 9.6 mm (reference point A), 14.0 mm (17.4%), 17,1 mm (34.8%) and 20.6 mm (52.1%), respectively. ‟Very short” SIJs were defined as articulation lines with a length ≤ 5 cm and concerned three cases. The respective distances between the SIJ and L5 were 11.3 mm (reference point A), 14.2 mm (17.4%), 16.3 mm (34.8%) and 18.9 mm (52.1%). Pelves including an SIJ with a length ≥ 6.5 cm (n = 6) were characterized as ‟very long” with corresponding distances of 9.8 mm (reference point A), 14.5 mm (17.4%), 16.5 mm (34.8%) and 18.9 mm (52.1%), indicating slightly more place directly at the linea terminalis level in ‟very short” SIJs.

### Measurements for the ventral ramus of L4

Measurements of the distance from reference point A along the linea terminalis to the ventral portion of L4 and the perpendicular distance at 17.4%, 34.8%, 52.1%, 69.5% and 86.3% were possible in 40, 40, 40, 40 and 13 cases, respectively. The corresponding distances were 7.1 mm, 10.6 mm, 13.9 mm, 17.5 mm, 20.7 mm and 21.8 mm (Fig. 2c).

The 13 ‟shorter” SIJs showed respective intervals of 7.5 mm (reference point A), 11.2 mm (17.4%), 15 mm (34.8%), 18.8 mm (52.1%) and 21.5 mm (69.5%). The ‟longer” SIJ subgroup showed corresponding distances of 7.4 mm (reference point A), 11.1 mm (17.4%), 14.1 mm (34.8%), 17.6 mm (52.1%), 19.9 mm (69.5%) and 21.8 mm (86.3%). ‟Very short” SIJs showed mean distances of 9.7 mm (reference point A), 12.2 mm (17.4%), 13.8 mm (34.8%), 16.4 mm (52.1%) and 18.8 mm (69.5%). The ‟very long” category had mean values of 7 mm (reference point A), 11.5 mm (17.4%), 13.8 mm (34.8%), 17.1 mm (52.1%), 18.5 mm (69.5%) and 20,4 mm (86.3%), respectively.

The mean distance between the point which was located 1 cm distal from reference point A inside the true pelvis and the lateral border of the LT was 0.3 mm (SD: 0.3; range 0–1.1).

### Analysis of sex and side differences

The superior aspect of the SIJ had a mean length of 57.8 mm (SD: 0.6; range 4.5–6.8) in males and of 58.5.mm (SD: 0.6; range 4.5–6.8) in females (p = 0.64). Male body donors were statistically significantly (p ≤ 0.001) older (mean: 87.7 years; SD: 4.8; range 75–92) when compared to females (mean: 77.9; SD: 8.2; range 56–96). The distances between the defined measurement points alongside the superior portion of the SIJ and L4 and L5 with regard to sex are displayed in Table 2. Here, no significant difference between the sexes could be evaluated for any of the distances.

**Table 2:** Sex differences of intervals between the ventral branches of L4/L5 and respective measurements points (Point A on the linea terminalis and 17.4%/34.8%/52.1%/69.5%/86.3% of the total length of the sacro-iliac joint).

	L4			L5		
	Males	Females	p-value	Males	Females	p-value
**Point A**						
Mean	6.5	7.9	0.06	8.5	9.7	0.27
SD	2.1	2.4		2.5	2.8	
Minimum	1.9	3.8		3.2	5.4	
Maximum	10.2	11.8		12.6	15.6	
**17.4%**						
Mean	10.2	11.1	0.54	12.9	13.4	0.96
SD	2.2	2.5		2.3	2.8	
Minimum	3.9	7.8		5.2	9.5	
Maximum	13.9	16.7		16.8	19.3	
**34.8%**						
Mean	13.7	14.1	0.98	16.7	17.0	0.76
SD	2.8	3.6		3.1	4.3	
Minimum	6.5	9.1		8.9	12.1	
Maximum	21.3	24.4		24.4	28.7	
**52.1%**						
Mean	17.6	17.4	0.54	20.1	19.6	0.17
SD	3.5	4.7		4.2	6.9	
Minimum	11.5	11.6		13.2	15.3	
Maximum	26.1	31.6		28	38.3	
**69.5%**						
Mean	20.9	20.3	0.44			
SD	4.5	6.1				
Minimum	15.1	14.1				
Maximum	34.8	38.9				
**86.3%**						
Mean	22.6	19.9	0.26			
SD	2.9	3.0				
Minimum	17.3	17.8				
Maximum	26.5	24.3				

Data are presented in millimeters.

The superior portion of the SIJ had a mean length of 5.9 mm (SD: 0.6; range 4.5–6.9) on the left and 5.7 mm (SD: 0.5; range 5–6.6) on the right side (p = 0.50). Distances between the measurement points and L4 and L5 concerning side are displayed in Table 3. No significant differences could be evaluated for any of the distances with regard to body side.

**Table 3:** Side differences of intervals between the ventral branches of L4/L5 and respective measurements points (Point A on the linea terminalis and 17.4%/34.8%/52.1%/69.5%/86.3% of the total length of the sacro-iliac joint).

	L4			L5		
	Left	Right	p-value	Left	Right	p-value
**Point A**						
Mean	6.8	7.3	0.45	8.9	9.0	0.79
SD	1.6	2.8		1.7	3.3	
Minimum	3.5	1.9		6	3.2	
Maximum	9.6	11.9		12.4	15.8	
**17.4%**						
Mean	10.7	10.4	0.75	13.3	12.9	0.62
SD	1.7	2.9		1.8	3.1	
Minimum	8.0	3.9		9.9	5.2	
Maximum	13.9	16.7		16.8	19.1	
**34.8%**						
Mean	14.7	13.1	0.23	17.4	16.2	0.48
SD	3.2	2.8		3.8	3.2	
Minimum	10.8	6.5		12.1	8.9	
Maximum	24.3	17.5		28.7	21.2	
**52.1%**						
Mean	18.3	16.7	0.34	21.2	18.6	0.20
SD	4.5	3.3		5.9	3.9	
Minimum	12.2	11.5		15.6	13.2	
Maximum	31.6	22.7		38.3	26.3	
**69.5%**						
Mean	21.9	19.5	0.07			
SD	5.3	4.8				
Minimum	16.6	14.1				
Maximum	38.9	34.8				
**86.3%**						
Mean	22.4	20.9	0.52			
SD	2.9	3.6				
Minimum	17.8	17.3				
Maximum	26.5	24.9				

Data are presented in millimeters.

## Discussion

The aim of the present anatomical study was to correlate the distance between the ventral rami of the fourth and fifth lumbar spinal nerves with the length of the anterior wall of the SIJ.

The superior wall of the SIJ had a mean length of 58.0 mm. Intervals between the anterior part of the joint and the lateral border of the ventral branch of L5 were at a mean of 8.9 mm (superior part of the fibrous capsule covering the SIJ at the intersection with the linea terminalis), 13.1 mm (17.4%), 16.8 mm (34.8%) and 19.9 mm (52.1%). The distance between the superior portion of the fibrous capsule covering the SIJ at the linea terminalis and the lateral border of L5 was increased in pelves with a ‟very short” (length ≤ 5 cm) SIJ (mean: 11.3 mm) when compared to those with a ‟very long” (≥ 6.5 cm) SIJ (mean: 9.8 mm). Regarding L4, the corresponding distances between the SIJ and the ventral branches’ lateral border were 7.1 mm (superior part of the fibrous capsule covering the SIJ at the intersection with the linea terminalis), 10.6 mm (17.4%), 13.9 mm (34.8%), 17.5 mm (52.1%), 20.7 mm (69.5%) and 21.8 mm (86.3%). Again, ‟very short” SIJs had longer (mean: 9.7 mm) distances between the reference point at the linea terminalis and the lateral border of the ventral branch of the nerve in comparison to the ‟very long” subgroup (mean: 7 mm). Concerning all evaluated data, no statistically significant sex or side differences could be observed.

In cases of anterior plate stabilization during pure SIJ dislocations or fracture dislocations, the LT is at risk, especially during surgical exposure of the superolateral aspect of the sacrum^7,13,14^.

Atlihan et al.^15^ performed anatomical dissections of the LT in 60 specimens to evaluate the nerves at risk during approaches to the anterior wall of the SIJ. The distance between the joint line and the lateral border of L5 was at a mean of 18.4 mm at the height of its exit point from the intervertebral foramen. At the linea terminalis, the interval between the LT and the SIJ was at a mean of 5.3 mm. In the present collective this was longer with means of 8.9 mm (L5) and 7.1 mm (L4). The length of the anterior wall of the SIJ was well comparable between Atlihan et al. (mean: 58.9 mm) and the present sample (mean: 58.0 mm).

Ebraheim et al.^7^ performed dissections and measurements on 10 embalmed and 6 fresh anatomical specimens. Authors reported mean distances of 23 mm and 26 mm to the ventral branches of L4 and L5 at the height of 4 cm proximal to the linea terminalis at the level of the anterior wall of the SIJ. At the junction between the false and true pelvis a mean distance of 10 mm for both structures was observed. In the current study, these values were at a mean of 8.9 mm (L5) und 7.1 mm (L4) at the height of the linea terminalis.

Bai et al.^9^ used 15 formalin-embalmed pelvic specimens for their anatomical study. Authors marked the highest point of the anterior wall of the SIJ as point A, its lowest point as point C (at the linea terminalis) and the midpoint of A and C as reference point B. The horizontal distances between these points and the lateral borders of the ventral branches of L4 and L5 were evaluated. Mean values were 21 mm (point A), 17 mm (point B) and 12 mm (point C) for L4 as well as 26 mm (point A), 22 mm (point B) and 15 mm (point C) for L5. For the current study, point B of Bai et al. is comparable to the measurement height of 52.1% of the total length of the SIJ and point C to our reference point A (superior portion of the SIJ capsule at the linea terminalis). Regarding L4, authors’ result (mean: 17 mm) is well comparable to our value (mean: 17.5 mm), whereas for L5 we evaluated a slightly lower mean (19.9 mm). At the linea terminalis, we evaluated smaller average distances for both, L4 (7.1 mm) and L5 (8.9 mm).

Differences of the intervals between the nerves and the SIJ may possibly be traced back to varying embalming methods.

Waikakul et al.^16^ evaluated 52 embalmed hemipelves regarding the branches of the lumbosacral plexus. With reference to the most anterior part of the SIJ, the union of the ventral branches of L4 and L5 to the LT was located above (11 specimens), directly on (12 specimens) or below (8 cases) this basing point. In our study, nearly all unions occurred inside the true pelvis at the anterior sacral surface.

Concerning sex differences, Ebraheim et al.^17^ dissected the LT bilaterally in twenty anatomical specimens. Authors reported the ventral branch of L5 as the thickest branch in males (mean: 4.4 mm) and the ventral portion of S1 as the thickest branch in females (mean: 4.3 mm). Though these data haven’t been evaluated in the present sample, we found no statistically significant differences between the sexes for any of our evaluated distances.

Pascal-Moussellard et al.^18^ reported a 5% affection of the ventral branch of L5 and the LT in sacral zone1 fractures according to the Denis classification. However, plate positioning and fixation at the linea terminalis is only possible in direct relation to these nerve roots. Only a direct subperiosteal dissection can avoid injury to these branches. In the ‟very small” pelves of our sample, the distance between the anterior wall of the SIJ and the lateral borders of L5 (mean: 11.3 mm) and L4 (mean: 9.7 mm) at the linea terminalis increased to acceptable values. At the level 1 cm (17.2% of SIJ length) proximal/posterior to point A this distance was ≥ 1 cm, which allows usually placement of one screw into one plate hole.

This given study is accompanied by the following limitations, which need to be addressed in the context of the here given results and interpretations. First, it has to be stated that the length of the SIJ has been measured as a straight distance between points A and B in an effort to simplify from the complex curvatures of the sacroiliac joint and adjacent substructures. This simplification will likely have resulted in marked distortion, which was however necessary to obtain comparable measurements. A further limitation has been introduced by the choice of the embalming technique for the tissues which were deployed for the measurements^11^. Modified Thiel’s solution^11^ includes a significant amount of osmotically active and fat dissolving chemicals. As a consequence, cell washout and tissue degradation as well as extensive degreasing are observed in the tissues. These effects have been exemplified in the tissues presented in the figures, showing a markedly different state of preservation. Given the nerve tissues investigated here consist of a significant amount of fat, the bias introduced by tissue distortion or shrinkage is unknown^19^. In consequence, further study comparing the LT with without the given embalming effects are necessary in near future. Also, we solely used anatomical geriatric specimens of Caucasian origin. Therefore, there might be a potential selection bias, as our findings might not align with morphometries obtained in other ethnicities or a younger cohort.

A safe zone of approximately 1 cm to 2 cm (anterior to posterior) is present on the sacral surface (lateral to medial) for safe fixation of plates during anterior plate stabilization of the SIJ. The results of our study indicate that pelves with a shorter dorsoventral diameter of the most superior part of the SIJ include more space for plate insertion.
